# Clinical characteristics and management of dupilumab-associated ocular surface disease in Japan

**DOI:** 10.1007/s10384-025-01299-9

**Published:** 2025-11-21

**Authors:** Jun Shoji, Nobuyuki Ebihara

**Affiliations:** 1 Division of Ophthalmology, Department of Visual Sciences, Nihon University School of Medicine, 30-1 Oyaguchi-Kamichou, Itabashi-ku, Tokyo 173-8610 Japan; 2 Department of Ophthalmology, Juntendo University Urayasu Hospital, Urayasu, Japan

**Keywords:** Atopic dermatitis, Conjunctival hyperemia, Dupilumab, Management framework, Ocular surface diseases

## Abstract

Dupilumab-associated ocular surface disease (DAOSD) is one of the most common adverse events associated with dupilumab (an anti-interleukin-4-receptor-alpha monoclonal antibody) during the treatment of patients with atopic dermatitis (AD). However, it rarely occurs in patients with bronchial asthma or chronic rhinosinusitis with nasal polyps. Adequate understanding of DAOSD is important for proper diagnosis and appropriate ophthalmic intervention. The aim of this review was to summarize and discuss the clinical characteristics and management of DAOSD in Japan. The pathogenesis of DAOSD can be explained by the dry eye disease, upregulated T helper 17 and 22 cells, and *Demodex* theories. The main symptoms of DAOSD are irritation/pain, redness, pruritus, discharge, and light sensitivity. Patients with AD and DAOSD as an adverse event develop various types of ocular surface disease, including blepharitis, blepharoconjunctivitis, conjunctivitis, keratoconjunctivitis, and keratitis. In ophthalmologic practice, to diagnose and treat DAOSD, physicians must understand the condition of the patient, make a differential diagnosis of conjunctivitis, determine concurrent dry eye, and assess DAOSD severity. Red flags for ophthalmologic intervention have been reported by organizations and institutions in various countries, which have highlighted the need for appropriate ophthalmologic intervention. Treatment of DAOSD involves topical treatments with artificial tears, antiallergic drugs, corticosteroids, and immunosuppressive drugs. In conclusion, patients with severe DAOSD require ophthalmologic intervention, and clinical collaboration between ophthalmologists and dermatologists is crucial for patients with AD during dupilumab treatment. This review can assist ophthalmologists in their daily practice and in their management of patients with DAOSD.

## Introduction

Dupilumab (Dupixent, Sanofi) is an antihuman interleukin (IL)-4 receptor α monoclonal antibody indicated for the treatment of moderate-to-severe atopic dermatitis (AD), moderate-to-severe bronchial asthma (BA) with type 2 inflammation, and chronic rhinosinusitis with nasal polyps (CRwNP) in adolescents and adults [1]. In Japan, the indications for use have been expanded to include prurigo nodularis and chronic spontaneous urticaria, and the age range for AD has been extended to include children aged ≥6 months.

Conjunctivitis is one of the most common adverse events associated with dupilumab treatment in patients with AD; however, it rarely occurs in patients with BA or CRwNP. A variety of ocular surface diseases that occur during dupilumab treatment have been reported in case series and clinical studies and are now termed dupilumab-induced ocular surface disease (DIOSD) [2–4], dupilumab-related ocular surface disorders (DROSD) [5], or dupilumab-associated ocular surface disease (DAOSD) [6, 7]. In this review, the term DAOSD is used for ophthalmologic adverse events associated with dupilumab. Ophthalmologists should be familiar with DAOSD including its pathophysiology, subjective symptoms, and objective findings to diagnose and treat it accurately. Additionally, clinical collaboration between ophthalmologists and dermatologists is crucial when administering dupilumab for the treatment of patients with AD. The aim of this review is to summarize and discuss the clinical characteristics and management of DAOSD in Japan to assist ophthalmologists in their daily practice. This review also emphasizes the perspective of ophthalmologists, which has been lacking until now. It collates information that will be useful in their daily practice.

## AD immunopathology and dupilumab

### Immunologic background of AD

AD is a pruritic eczematous dermatitis; its symptoms chronically fluctuate with remission and relapse. Most individuals with AD have atopic diathesis [8]. The pathogenesis of AD is multifactorial, driven by a complex interplay between environmental factors, epidermal barrier defects, immune response dysregulation, and genetic polymorphisms [9].

The clinical characteristics of AD vary depending on age, disease stage, race or ethnic group, and geographic location. The acute skin lesions are characterized by papules, papulovesicles, edema, crusting, and scaling, with hyperpigmentation or hypopigmentation after healing. In severe AD, areas of eczema coalesce into larger regions of generalized redness of the skin (erythroderma). Regarding immune response dysregulation, AD is classified as a T helper (Th) 2-type immune disorder. IL-4, IL-5, and IL-13 are crucial Th2 cytokines related to the type 2 inflammation that occurs in AD-affected areas, and also induce itching of the skin. Type 2 inflammation involves immunoglobulin E (IgE)-dependent adaptive allergic reactions dependent on Th2 cells and IgE-independent innate allergic reactions dependent on innate lymphoid cell group 2 (ILC2) cells. Regarding adaptive allergic reactions, AD has been classified as a Th2 skin disease, but it is now understood to be a much more heterogeneous disease, involving additional activation of the Th22, Th17/IL-23, and Th1 cytokine pathways, depending on the disease subtype [10–12].

Furthermore, innate allergic reactions are also involved in the pathogenesis of AD. Thymic stromal lymphopoietin (TSLP) is a cytokine related to innate immunity and is highly expressed in the skin epidermal cells of patients with AD [13]. TSLP in the skin induces a Th2 response by acting on CD11c^+^ dendritic cells to produce Th2 chemokines including CC chemokine ligand (CCL) 17/thymus, activation-regulated chemokines (TARC), and CCL22/macrophage-derived chemokines [13]. It induces allergic reactions by increasing the production of IL-4 via basophils [14]. Additionally, through skin scraping, keratinocytes produce the alarmin cytokines IL-33 and IL-25, which are involved in eosinophilic inflammation (type 2 inflammation) by increasing the production of IL-5 and IL-13 from ILC2 [15].

### Dupilumab: mechanism of action and efficacy

**Fig. 1:**
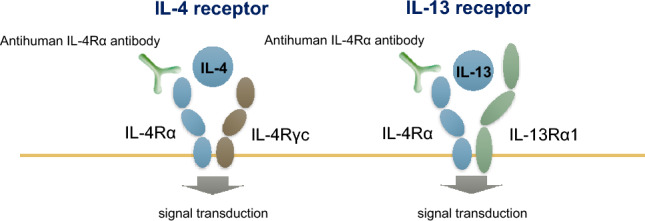
Pharmacologic action of dupilumab. Dupilumab, an antihuman interleukin (IL)-4 receptor α antibody, acts on the IL-4 receptor α to block binding to the IL-4 and IL-13 receptors. *IL4R* interleukin-4 receptor

Dupilumab is a human monoclonal IgG4 antibody drug used in antibody therapy targeting IL-4 and IL-13. The alpha-chain of the IL-4 receptor is common to the IL-4 and IL-13 receptors (Fig. 1). Dupilumab binds to the common alpha-chain of the IL-4 receptor, inhibiting signaling mediated by both IL-4 and IL-13 and reducing type 2 inflammation [16]. Clinically, the recommended starting dose for adults and children aged ≥12 years is 600 mg by subcutaneous injection at week 0, followed by a dose of 300 mg every 2 weeks.

The efficacy and safety profile of dupilumab in monotherapy and in combination with topical corticosteroids of moderate potency has been demonstrated in 3 randomized, double-blind, placebo-controlled clinical trials (LIBERTY AD SOLO-1, LIBERTY AD SOLO-2, and LIBERTY AD CHRONOS) [17, 18]. Randomized, placebo-controlled phase III studies showed that dupilumab improves lesion resolution, pruritus, sleep quality, and quality of life when compared with placebo. A systematic review and meta-analysis of real-world evidence for dupilumab analyzed 22 studies and 3303 patients with AD [19]. After 16 weeks of dupilumab therapy, the pooled proportions of patients achieving a 50%, 75%, and 90% Eczema Area and Severity Index score improvement were 85.1%, 59.8%, and 26.8%, respectively.

### Effects of dupilumab on allergic conjunctival diseases

Dupilumab, although used off-label in Japan, has been reported to be effective in the treatment of allergic conjunctival diseases. Fukuda and colleagues reported on a patient with atopic keratoconjunctivitis (AKC) whose giant papillae disappeared during dupilumab treatment for AD [20]. This suggests that the major pathogenesis of giant papillae may be type 2 inflammation. Another case report presented a milder clinical presentation of AKC [21].

The efficacy of dupilumab in treating vernal keratoconjunctivitis (VKC) is unknown; however, a case series study demonstrated its effectiveness for treating recurrent refractory VKC [22]. All patients with VKC showed subjective symptom improvement, resolution of the shield ulcer, reduction of conjunctival hyperemia, and complete resolution of giant papillae after dupilumab treatment [22].

## DAOSD

### Epidemiology

Conjunctivitis is one of the common adverse events associated with dupilumab treatment for AD, with a reported incidence of 8.6% to 22.1% in phase 2b and 3 dupilumab clinical trials (including SOLO1, SOLO2, CHRONOS, and CAFÉ) (Table 1) [18, 23–32]. The frequency of conjunctivitis was significantly lower with dupilumab treatment in patients with asthma and chronic sinusitis than in patients with AD and did not differ from the placebo group in 1 clinical trial [23]. Therefore, DAOSD is considered an adverse event specific to patients with AD.

**Table 1:** Incidence of conjunctivitis after administration of dupilumab in clinical trials of dupilumab for atopic dermatitis

Phase	Trial name	Eligible patients	Treatment duration	Treatment group	Onset of conjunctivitis: No. of cases (%)	
					Placebo group	Dupilumab group
III	SOLO1^a^	Patients with ≥ moderate AD and inadequate response to topical steroids	16 weeks	Placebo group / dupilumab group	2/222 (1.0%)	18/447 (4.0%)
III	CHRONOS^b^	Patients with moderate-to-severe AD	52 weeks	Placebo + TCS / dupilumab (300 mg weekly or biweekly) + TCS	25/315 (7.9%)	76/425 (17.9%)
III	LIBERTY AD OLE^c^	Patients with moderate-to-severe AD who had completed a previous phase I–III study	4 years	Dupilumab group (open-label)	–	276/2,677 (10.3%)
III	LIBERTY AD PRESCHOOL^d^	Pediatric patients with moderate-to-severe AD aged ≥6 months or <6 years	16 weeks	Placebo group + low-dose TCS / dupilumab group + low-dose TCS	0/78 (0%)	4/83 (4.8%)
III	LIBERTY AD PEDS^e^	Critically ill pediatric patients with AD aged 6–11 years	16 weeks	Placebo + TCS dupilumab + TCS	5/120 (4.2%)	26/242 (10.7%)
III	LIBERTY AD ADOL^f^	Pediatric patients with moderate-to-severe disease AD aged 12–17 years	16 weeks	Placebo group / dupilumab group	4/85 (4.7%)	17/165 (10.3%)
II	Japan Pediatric AD Phase III Study^g^	Japanese patients with moderate-to-severe AD aged 6 months to 17 years	16 weeks	Placebo + TCS / dupilumab + TCS	0/32 (0%)	4/30 (13.3%)

*AD* atopic dermatitis, *TCS* topical steroids

^a^Simpson EL et al. N Engl J Med. 2016;375:2335–48 (Reference 24)

^b^Blauvelt A et al. Lancet 2017;389(10086):2287–303 (Reference 18)

^c^Beck LA et al. Am J Clin Dermatol. 2022;23:393–408 (Reference 28)

^d^Paller AS et al. Lancet 2022;400(10356):908–19 (Reference 29)

^e^Paller AS et al. J Am Acad Dermatol. 2020;83:1282–93 (Reference 30)

^f^Simpson EL et al. JAMA Dermatol. 2020;156:44–56 (Reference 31)

^g^Ebisawa M et al. Allergol Int. 2024;73:532–42 (Reference 32)

Hirai and colleagues analyzed adverse drug reactions (ADRs) associated with dupilumab using data from the World Health Organization’s VigiBase [33]. Ocular ADRs were ranked fourth in terms of organ level, and dry eye, blepharitis, and conjunctivitis were significantly associated with ADRs in patients aged ≤44 years.

Various published data have been published on the incidence of conjunctivitis during dupilumab use in real-world clinical settings [34–41] (Table 2). In Japan, a 1-year interim analysis from a postmarketing surveillance of dupilumab for AD was conducted, and the results of 599 patients with AD were published [34]. Ninety-eight patients with allergic conjunctivitis (16.4%) were reported at the time of registration, and during the mean (± standard deviation) observation period of 39.5 ± 27.8 weeks, 94 patients (15.7%) had concomitant adverse events, including 40 cases (6.7%) of conjunctivitis and 30 cases (5.0%) of allergic conjunctivitis. Nettis and colleagues investigated 72 cases of AD treated with dupilumab and reported the development of conjunctivitis in 29 cases (40.3%) [42]. The severities of these 29 cases were as follows: 3 severe cases (10.3%), 8 moderate cases (27.6%), and 18 mild cases (62%). Most of the cases of conjunctivitis that occurred with dupilumab treatment were mild-to-moderate, and 19 of the 29 cases (65.5%) improved within 16 weeks.

**Table 2:** Onset of conjunctivitis after administration of dupilumab in patients with atopic dermatitis (real-world clinical settings)

Country	Registry/Exam name	Eligibility	Observation period	Number of cases	Ophthalmologist consultation	Therapy
Japan	Survey on Specific Use Results (Interim Report)^a^	Patients with AD receiving dupilumab (n = 599)	Mean (±SD) observation period 39.5 ± 27.8 weeks	Conjunctivitis: 6.7% (40/599) Allergic conjunctivitis: 5.0% (30/599)	Unknown	
Netherlands	BioDay registry^b^	Patients receiving dupilumab (n = 167)	1 year	Conjunctivitis: 39.5% (66/167)	33/66 patients were examined by an ophthalmologist and detailed ophthalmologic findings were reported	Artificial tears 78.8% (26/33), tacrolimus ointment 75.8% (25/33), steroid eye drops 72.7% (24/33), antihistamine eye drops 42.4% (14/33), etc
Netherlands	Multicenter prospective observational study^c^	Patients with AD receiving dupilumab (n = 95)	12 weeks	Ocular symptoms including redness and itching: 62.1% (59/95)	16 patients were examined by an ophthalmologist; 9 were diagnosed with conjunctivitis (allergic keratin), 2 with blepharitis, and 2 with xerophthalmia	Artificial tears, antihistamine eye drops, fluorometholone eye drops, tacrolimus ointment
France	GREAT registry^d^	Patients with AD receiving dupilumab (n = 241)	3 months	Conjunctivitis: 38.2% (84/220)	Diagnosis of conjunctivitis is made by a dermatologist. Of the 39 patients who received ophthalmologic examinations, 32 were also diagnosed with conjunctivitis by an ophthalmologist	
France	GREAT registry^e^	Patients with AD receiving dupilumab (n = 181)	16 weeks	At 16 weeks, 34 ophthalmologists diagnosed blepharoconjunctivitis, 32 of which were new. Others: 14 cases of keratitis, 11 cases of dry eye, and 6 cases of conjunctival fibrosis	Observation was performed by a dermatologist and an ophthalmologist during baseline and treatment	
Germany	TREATgermany registry^f^	Patients with AD receiving dupilumab (n = 137)	3 months, 6 months	Conjunctivitis: 13.3% after 3 months (14/105), 22.6% after 6 months (12/53)		
Germany	TREATgermany registry^g^	Patients with AD receiving dupilumab (n = 369)	December 2017–July 2021	Dupilumab-related ocular symptoms 29.8%, conjunctivitis 20.7%		
Netherlands	BioDay registry^h^	Patients receiving dupilumab (1223 cases)	October 2017– September 2022	Conjunctivitis 33.7% (412/1223 cases), 3.4% (42/1223 patients) discontinued dupilumab because of dupilumab-associated conjunctivitis		Anti-inflammatory eye drops (moderate-to-severe conjunctivitis)

*AD* atopic dermatitis

^1^Saeki H et al. J Cutan Immunol Allergy 2023;6:78–87 (Reference 34)

^2^Achten R et al. J Allergy Clin Immunol Pract. 2021;9:1389–92 (Reference 35)

^3^de Wijs LEM et al. Br J Dermatol. 2020;182:418–26 (Reference 36)

^4^Faiz S et al. J Am Acad Dermatol. 2019;81:143–51 (Reference 37)

^5^Costedoat I, et al. J Eur Acad Dermatol Venereol. 2023;37:1056–63 (Reference 38)

^6^Abraham S et al. Br J Dermatol. 2020;183:382–84 (Reference 39)

^7^Stölzl D, et al. Br J Dermatol. 2022;187:1022–24 (Reference 40)

^8^Zhang J et al. J Am Acad Dermatol. 2024;91:300–11 (Reference 41)

### Clinical presentation and subtypes

The clinical presentation of ocular surface adverse events has been reported by allergologists, dermatologists, and ophthalmologists as dupilumab-associated conjunctivitis, DIOSD [2–4], DROSD [5], and DAOSD [6, 7], according to the diverse forms and severities of the ophthalmologic findings. In particular, detailed ocular surface disease types and clinical findings, including follicular conjunctivitis [43, 44], cicatrizing conjunctivitis [45, 46], blepharoconjunctivitis [47], keratitis [44, 48], episcleritis [49], and dry eye disease [50], have been reported by ophthalmologists. Rare cases of ocular diseases, including corneal perforation [51] and uveitis, have also been reported [52, 53] (Table 3).

**Table 3:** Clinical phenotypes of dupilumab-associated ocular surface disease

Clinical phenotype	Study references
*Conjunctivitis*	
Giant papillary conjunctivitis	81, 82
Follicular conjunctivitis	43, 44
Cicatricial conjunctivitis	45, 46
Dry eye	50
Superior limbic keratoconjunctivitis-like conjunctivitis	78
*Blepharitis*	
Blepharoconjunctivitis	47
Meibomian gland dysfunction	47, 79
*Keratitis*	
Superficial punctate keratitis	50
Marginal keratitis	49, 78
Corneal perforation	51
*Episcleritis*	
Sectorial episcleritis	49
*Uveitis*	52, 53

### Proposed pathophysiologic mechanisms

There are several DAOSD pathogenesis theories, including the dry eye disease, upregulated Th17 and Th22 cells (inflammation derived from Th1, Th17, and Th22 responses), and *Demodex* theories, described below.

### *Dry eye and goblet cell deficiency*

Patients with DAOSD show signs of dry eye assessed using clinical dry eye tests, including the Schirmer test, tear break-up time, and fluorescein staining. Furthermore, a pathologic study of patients with DAOSD reported a decrease in goblet cells in the conjunctiva [54]. The Th1-related and mucin deficiency dry eye theories are being investigated as potential explanations of dry eye in patients with DAOSD.

According to the Th1/Th2 balance theory [55], Th2-associated diseases include allergic diseases, whilst Th1-associated diseases include autoimmune diseases. Therefore, when dupilumab, which suppresses the Th2 cytokines IL-4 and IL-13, is administered, the immune response becomes Th1-dominant. This causes an immune response similar to that seen in Sjögren syndrome, and consequently dry eye develops. A pathologic study of conjunctival biopsies from patients with DAOSD reported that conjunctival tissues with increased subepithelial cell infiltration had a high expression of interferon-gamma (IFN-γ), tumor necrosis factor-alpha (TNF-α), IL-10, and IL-17A [56]. This indicates the possibility of a Th1-skewing effect of dupilumab through blockage of both IL-4 and IL-13 signaling in immune cells, and is similar to the immune responses of Sjögren syndrome, in which goblet cells decrease owing to IFN-γ and TNF-α, with dry eye subsequently developing [57].

The mucin deficiency theory suggests that DAOSD develops in association with mucin-deficient dry eye because the effect of IL-13, which induces goblet cell differentiation, is suppressed by dupilumab, resulting in mucin deficiency on the ocular surface [6, 54, 58]. IL-4 and IL-13 both contribute to the homeostasis of human conjunctival goblet cells in vitro [59]. A reduction in goblet cells in the conjunctiva and dupilumab-associated mucin deficiency due to decreased MUC5AC in tears has been reported in patients with AD during dupilumab use [6, 58]. In contrast, the expression of gel-forming mucin, including MUC5AC, in the conjunctiva is also decreased in patients with AKC not treated with dupilumab [60, 61]. Maudinet and colleagues reported 2 types of conjunctivitis that develop in patients with AD during dupilumab use: “nonspecific conjunctivitis associated with inferior punctuated corneal epithelial lesions,” which is associated with evaporative dry eye, and “severe dupilumab-induced follicular conjunctivitis,” which is not associated with dry eye [44]. Therefore, at least 2 forms of DAOSD may exist, a mild form with dry eye and a severe form without dry eye, and the pathogenesis of conjunctivitis before dupilumab administration may play a role in the development of DAOSD. Dry eye disease has been shown to be a potential pathologic condition associated with DAOSD; however, this does not account for the fact that DAOSD often occurs in patients with AD, but not in those with BA or CRwNP.

### *Th17/Th22-mediated inflammation*

In addition to type 2 inflammation, Th1, Th17, and Th22 cells are involved in the immunologic pathogenesis of AD; these immunologic mechanisms are complicated, especially in chronic skin lesions of adult patients. Such immunologic backgrounds of patients may be the reason why DAOSD is more likely to develop in adults with AD.

Th17 cells comprise an IL-17A/IL-17F/IL-22-secreting CD4-positive T cell subset that plays a key role in the defense against opportunistic fungal or bacterial pathogens but may also participate in autoimmune and allergic diseases [62]. Autoimmune diseases and allergic diseases are Th1- and Th2-associated diseases, respectively; however, Th17 may be involved in the development of both diseases. Additionally, IL-17A, a cytokine produced by Th17, may be involved in neutrophil inflammation of the airways in patients with BA [63, 64]. In patients with AD, IL-17 mRNA expression and the number of IL-17-positive cells are increased in the acute lesions of AD [65]. Furthermore, more prominent Th17 activation was observed in the blood and acute AD skin lesions of Asian patients than in those of European-American patients [66]. The immunologic background of patients with Th17 reactions in combination with type 2 inflammation, resulting in a mixed pattern of eosinophilic-neutrophilic inflammation, may be the reason for the variety of clinical manifestations and severity of DAOSD. Thormann and colleagues compared the cytokine profiles in tears of patients with AD, with and without conjunctivitis, and patients without AD during dupilumab treatment [66]. In patients with AD and DAOSD, the cytokine profiles in conjunctival tissues changed from Th2/Th17- to Th1/Th17-type responses [66].

Th22 cells are CD4-positive T cells that produce IL-22, but not IFN-γ, IL-4, or IL-17 [67]. In dermatologic diseases, Th22 cells are often infiltrated in the skin lesions of psoriasis and AD in the chronic phase [68, 69]. Th22 cells in the skin lesions of patients with AD are more frequent than in those of patients with psoriasis [69]. Additionally, IL-22 is mainly produced by Th17 and Th22 [70, 71], and high levels of IL-22 have been found in the skin and serum of patients with acute and chronic moderate-to-severe AD [71]. Furthermore, IL-22 correlates with TARC and is a useful biomarker of AD [72]. Adachi and colleagues examined cytokine-related mRNA expression levels on the ocular surface in patients with DAOSD [73]. IL-22 mRNA expression levels were significantly higher in patients with DAOSD than in those with AKC or those receiving dupilumab treatment for AD without conjunctivitis. Furthermore, IL-22 mRNA expression levels on the ocular surface were proposed as a useful biomarker of DAOSD [73]. Therefore, the administration of dupilumab can suppress the Th2 cytokines IL-4 and IL-13, resulting in an immunologic imbalance between Th1, Th2, Th17, and Th22, and causing Th22-related conjunctivitis.

In addition to DAOSD, dupilumab-associated head and neck dermatitis (DAHND) is another adverse event associated with dupilumab treatment, occurring in up to 10% of patients with AD. Bangert and colleagues profiled punch biopsies from patients with DAHND using single-cell RNA sequencing and compared the data with those of untreated AD patients and healthy control skin [74]. The findings revealed considerable increases in type 22-associated inflammation, especially in oligoclonally expanded T cells, accompanied by enhanced keratinocyte activation and IL-22 receptor upregulation. Therefore, the enhancement of Th22 responses in the lesions of DAOSD and DAHND, which are adverse events of dupilumab therapy, may be the result of disruption to the Th2/Th22 balance due to Th2 response suppression by dupilumab in AD lesions.

### *Demodex-associated inflammation*

Some patients with AD who receive dupilumab treatment develop rosacea-like dermatitis in the affected skin areas [75]. IL-4Rα knockout mice show an increase in *Demodex* in the skin hair follicles, and the occurrence of rosacea-like dermatitis [76]. The characteristic pathologic findings seen in the lesions of rosacea-like dermatitis include a coexistence of type 2 and Th17/Th22 inflammations, in addition to increased *Demodex* [76]. The similarities between the rosacea-like dermatitis in IL-4Rα knockout mice and that during dupilumab administration in patients with AD are being investigated. Abnormal immune responses associated with an increase of *Demodex* may change the skin lesions of AD. *Demodex* in the eyelashes is thought to be one of the triggers of blepharitis, and the potential involvement of this in the pathology of DAOSD is also being investigated [77]. Therefore, consideration of the similarities and differences between DAOSD and rosacea keratoconjunctivitis is also required.

## Clinical manifestations of DAOSD

### Subjective symptoms

Bohner and colleagues identified 5 subjective ocular symptoms that are frequently reported in patients with DAOSD: irritation/pain, redness, pruritus, discharge, and light sensitivity [7] (Table 4). Tauqeer and colleagues found that the most commonly reported ocular symptoms were redness (63%), tearing (47%), and itching (37%); other complaints included irritation, blurred vision, photophobia, discharge, foreign body sensation, and swelling [78]. Therefore, DAOSD is an ocular surface disease characterized by redness; however, it has no specific subjective symptoms.

**Table 4:** Subjective symptoms of dupilumab-associated ocular surface disease

Subjective symptom	Bohner et al 7	Tauqeer et al 78
Redness	24/29 (82.8%)	63%
Pain/irritation	28/29 (96.6%)	
Pruritus/itching	6/29 (62.1%)	37%
Discharge	18/29 (62.1%)	47%
Watering, tearing		
Light sensitivity	6/29 (20.7%)	
Swelling	3/29 (10.3%)	

### Objective ocular findings

The diagnosis of DAOSD requires a detailed examination of the ophthalmic clinical findings. This is due to the difficulty in distinguishing DAOSD from other ocular surface diseases including AKC, which occurs as an ocular complication of AD, and other infectious and noninfectious forms of keratoconjunctivitis. Characteristic ophthalmologic findings of DAOSD should be confirmed in the eyelid, palpebral conjunctiva, bulbar conjunctiva, limbus, and cornea.

### *Eyelid and meibomian gland involvement*

Blepharitis and blepharoconjunctivitis are among the ocular complications of AD. However, blepharitis may be exacerbated in patients with AD undergoing dupilumab therapy. Abnormalities in the meibomian glands are detected through clinical findings of meibomian gland dysfunction and morphologic abnormalities observed via meibography [47, 79, 80].

**Fig. 2:**
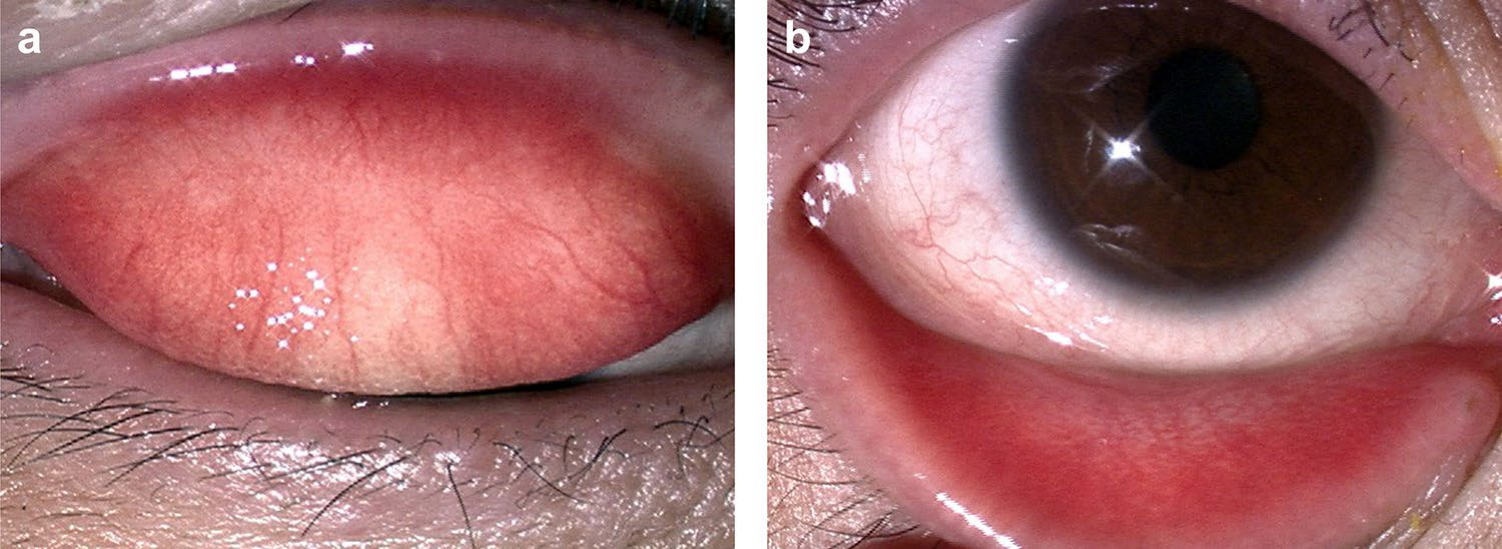
Conjunctival and corneal findings of mild-type dupilumab‐associated ocular surface disease. **a** Palpebral conjunctiva shows a fine reticular hyperemia and papillary formation. **b** Bulbar conjunctiva shows hyperemia with mild edema; however, these are not specific findings

**Fig. 3:**
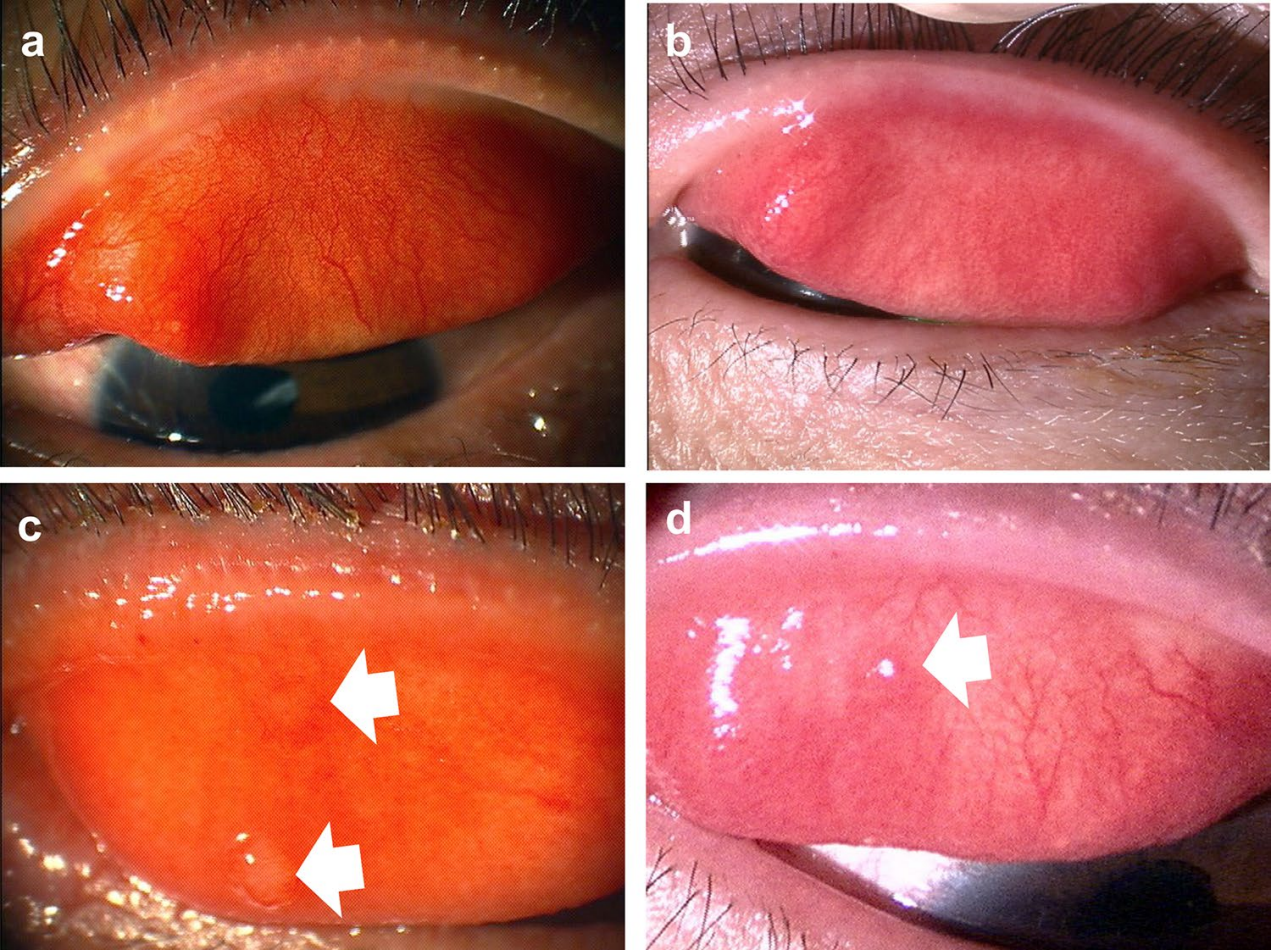
Clinically characteristic palpebral conjunctival findings of patients with dupilumab‐associated ocular surface disease. **a** Papillary formation with severe conjunctival hyperemia and swelling (velvety appearance). Plugging of the meibomian glands of the lid margin is visible at numerous locations. **b** Velvety appearance without plugging. **c, d** Giant papillae-like conjunctival proliferative lesions (arrow)

The main clinical findings of patients with blepharoconjunctivitis during dupilumab treatment are posterior blepharitis with meibomian gland dysfunction (Figs. 2 and 3a), edematous eyelids with multiple chalazia, significant papillary conjunctivitis, and evaporative dry eye on slit-lamp examination [47, 80]. Further clinical findings of these patients include loss of meibomian glands under meibographic examination, and cell infiltration, not only in the cornea and palpebral conjunctiva, but also in the meibomian glands, via confocal microscopic examination [79]. Therefore, the use of dupilumab may cause inflammation, not only in the conjunctiva and cornea, but also in the meibomian glands, and may disrupt the homeostasis of the ocular surface.

### *Conjunctival changes and papillary reactions*

In DAOSD, papillary proliferation with a velvety appearance in the palpebral conjunctiva is a representative clinical finding. This may or may not be accompanied by meibomitis (Fig. 3a, b), although the difference in conjunctivitis severity between the two is unclear. Conjunctival hyperemia, conjunctival swelling, and papillary proliferation, which are the major findings of the palpebral conjunctiva, are among the findings used to determine the severity of DAOSD. Giant papillae are also observed in the palpebral conjunctiva (Fig. 3c, d). However, giant papillae similar to vernal keratoconjunctivitis (VKC) and AKC are less frequently expressed, and are morphologically different from those of VKC and AKC [80, 81]. The atypical findings of giant papillae reported in patients with DAOSD comprise “lesion protruding from the upper fornix” and “giant papillae in the lower tarsal conjunctiva” [81, 82]. Therefore, giant papillae-like conjunctival lesions and flattened giant papillae that develop in patients receiving dupilumab therapy can be a useful factor for differential diagnosis [78].

### *Conjunctiva (bulbar and limbal)*

**Fig. 4:**
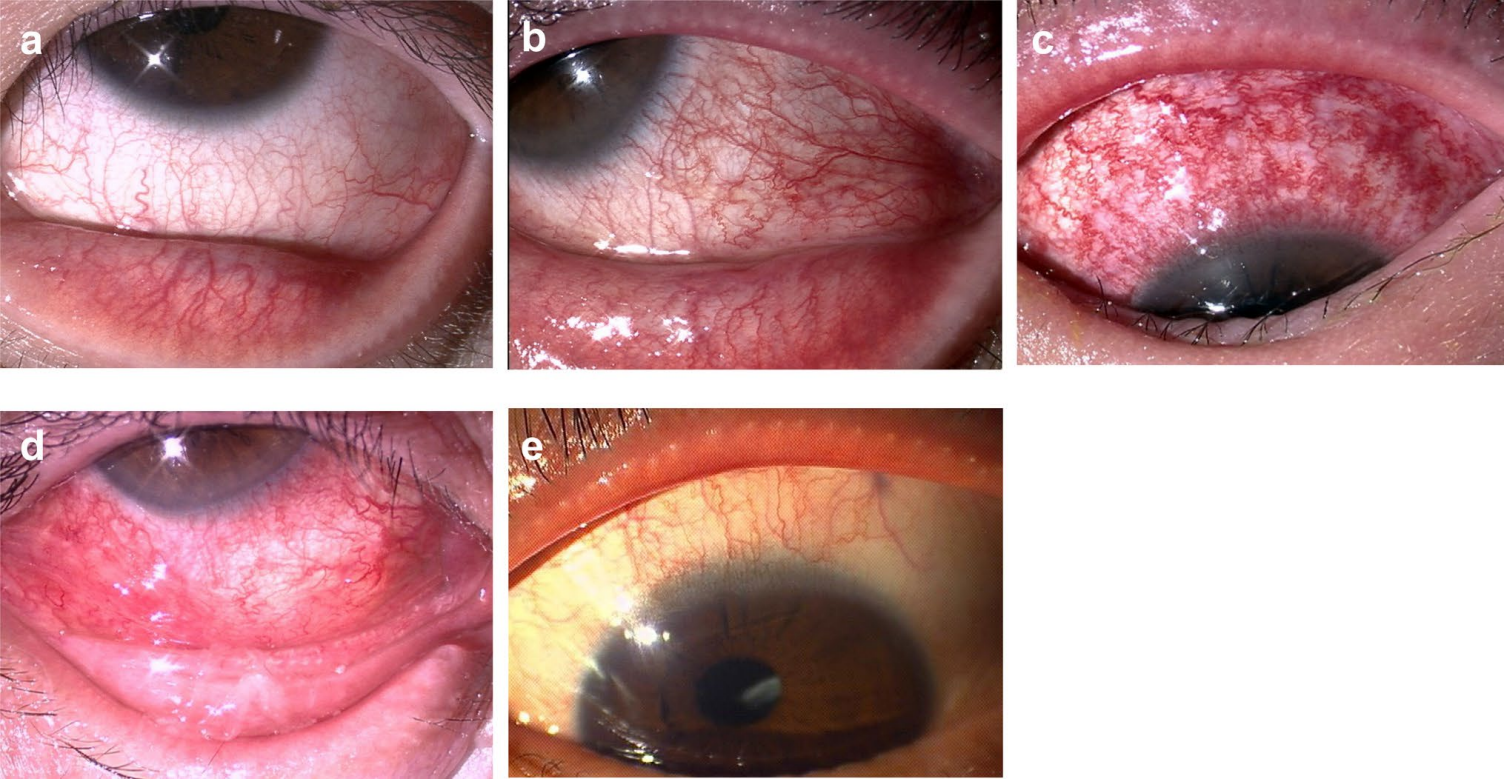
Clinically characteristic bulbar conjunctival findings of patients with dupilumab‐associated ocular surface disease. **a–c** Hyperemia in bulbar conjunctiva of **a** mild, **b** moderate, and **c** severe cases. **d** Episcleritis-like appearance in bulbar conjunctiva. **e** Superior limbic keratoconjunctivitis-like appearance in superior bulbar conjunctiva

Hyperemia in the bulbar conjunctiva is the most crucial clinical finding in patients with DAOSD. The degree of hyperemia varies from mild to severe (Fig. 4a–c), and regional differences exist such as whole bulbar conjunctiva and partial intensity in the upper or lower bulbar conjunctiva. In previous studies, these findings have been referred to as hyperemia with a red braid-like appearance for whole bulbar severe hyperemia [73] (Fig. 4c), episcleritis-like lower hyperemia for lower bulbar severe hyperemia [49] (Fig. 4d), and superior limbic keratoconjunctivitis-like upper conjunctival hyperemia for upper bulbar local hyperemia [78] (Fig. 4e). However, the pathologic conditions implied by these differences in hyperemia clinical findings are not fully understood.

**Fig. 5:**
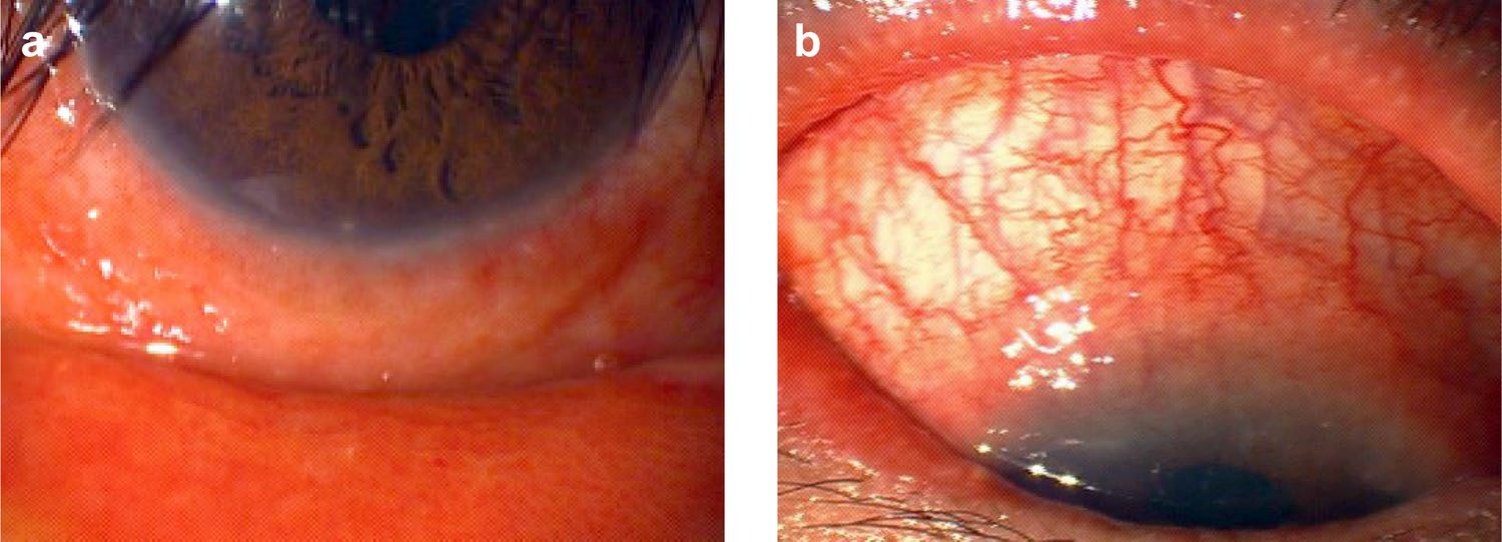
Clinically characteristic limbal findings of patients with dupilumab‐associated ocular surface disease. **a** Limbal swelling with peripheral corneal infiltration. White dot opacity is observed in the peripheral subepithelial cornea. **b** Severe limbal swelling with episcleritis-like hyperemia of the bulbar conjunctiva

The characteristic limbal findings observed in DAOSD are limbal swelling with peripheral corneal infiltration, similar to the limbal findings associated with scleritis or episcleritis (Fig. 5a, b). These findings expressed in conjunctival limbs have been reported under terms such as “perilimbal nodules,” “limbal follicles,” and “limbal infiltration” [44, 49, 78]. In contrast, AKC and VKC show limbal swelling with Horner–Trantas dots, which is thought to be caused by type 2 inflammation and eosinophilic infiltration [82]. Therefore, it is important for ophthalmologists to clarify the differences between these 2 types of limbal swelling.

### *Cornea*

**Fig. 6:**
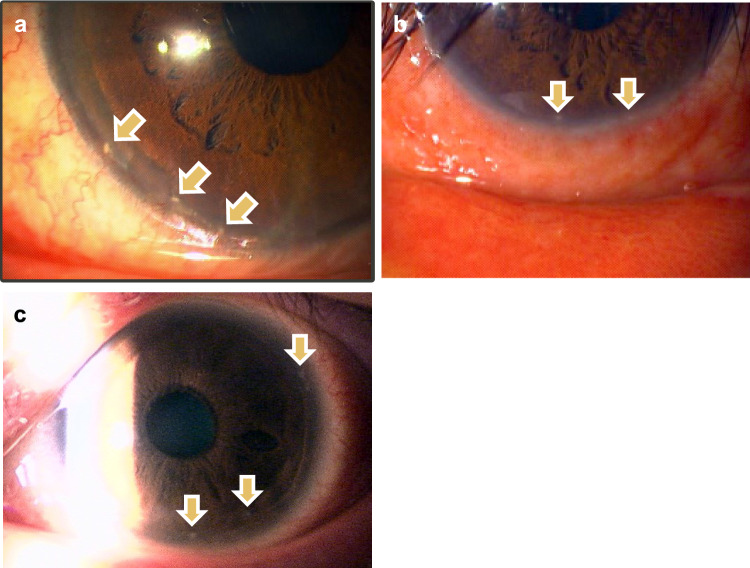
Clinically characteristic corneal findings of patients with dupilumab‐associated ocular surface disease. **a** White dots (arrow) in the peripheral cornea. **b** White dots (arrow) with corneal infiltration in the marginal cornea. **c** Scleral scatter view of white dots (arrow) with corneal infiltration in the peripheral cornea

In severe DAOSD, in addition to limbal swelling, the adjacent peripheral cornea is accompanied by infiltrative corneal stromal opacity [49, 78]. When compared with VKC, the corneal findings of DAOSD are characterized by a strong infiltrative opacity of the corneal stroma, and in some cases, small white-dot opacity occurs concurrently [78] (Fig. 6a–c). In cases where dry eye is also present, the cornea and conjunctiva may show findings similar to those of Sjögren syndrome. Therefore, corneal and conjunctival epithelial disorders should be checked by use of fluorescein staining during DAOSD examination.

## DAOSD diagnostic approaches

### Clinical scoring systems

Several methods for evaluating the clinical findings of DAOSD are available. Achten and colleagues evaluated 8 assessment items—blepharitis, meibomian gland dysfunction, tarsal conjunctivitis, bulbar conjunctivitis, limbitis, limbal vascularization, corneal punctate, and hurricane pattern—on a 4-point scale (none, mild, moderate, and severe) called the Utrecht Ophthalmic Inflammatory and Allergic disease ocular surface score [35]. Adachi and colleagues used the DAOSD grading scale, which consists of 4 categories—papillary proliferation in the palpebral conjunctiva, giant papillae, hyperemia of the bulbar conjunctiva, and limbal infiltration—to assess conjunctivitis severity in patients with DAOSD [73]. The DAOSD grading scale lists “red braid-like appearance” and “corneal white dots or infiltrates” as characteristic findings in severe cases. These clinical scores may be useful tools for objective assessment of the severity of DAOSD and evaluation of treatment efficacy. In addition, severity can be determined by subjective symptom scores [7].

### Tear and ocular surface biomarkers

In the ophthalmologic diagnosis of ocular surface diseases, the tear test and ocular surface test using biomarkers may be useful for diagnosis, determination of disease severity, and evaluation of treatment efficacy [83]. Several studies have attempted to diagnose DAOSD by use of tear and ocular surface test biomarkers.

Regarding tear tests, numerous studies have investigated changes in cytokines and chemokines in tear fluid. IL-33, an alarmin released via necrosis of epithelial cells such as conjunctival epithelial cells, is a potential predictive factor because it increases in tear fluid after DAOSD onset [84]. Tear fluid levels of AD-related severity biomarkers, including IL-22, TARC, and periostin, in patients with moderate-to-severe ocular surface disease decreased during dupilumab treatment; however, the tear fluid levels of these biomarkers in patients with moderate-to-severe ocular surface disease were slightly higher than those in patients with no or mild ocular surface disease [85]. Additionally, no specific differences in Th1- or Th17-associated biomarkers in tear fluid have been reported during dupilumab treatment [85]. In contrast, inflammatory markers in the tear fluid of patients with AD on dupilumab treatment are characterized by a shift from Th2/Th17 towards Th1/Th17 inflammation [66].

Regarding ocular surface tests using impression cytology and quantitative polymerase chain reaction, Adachi and colleagues reported cases of DAOSD in which IL-8 mRNA expression was elevated in the ocular surface test and suggested that patients with DAOSD may develop conjunctivitis because of a combination of eosinophil inflammation caused by AD and IL-8-induced neutrophil inflammation [86]. Additionally, these authors showed that when IL-22 was used as a biomarker in the ocular surface test, the sensitivity and specificity for diagnosing DAOSD were 70% and 100%, respectively, highlighting it as a useful biomarker [73].

### Conjunctival cytology and cultures

**Fig. 7:**
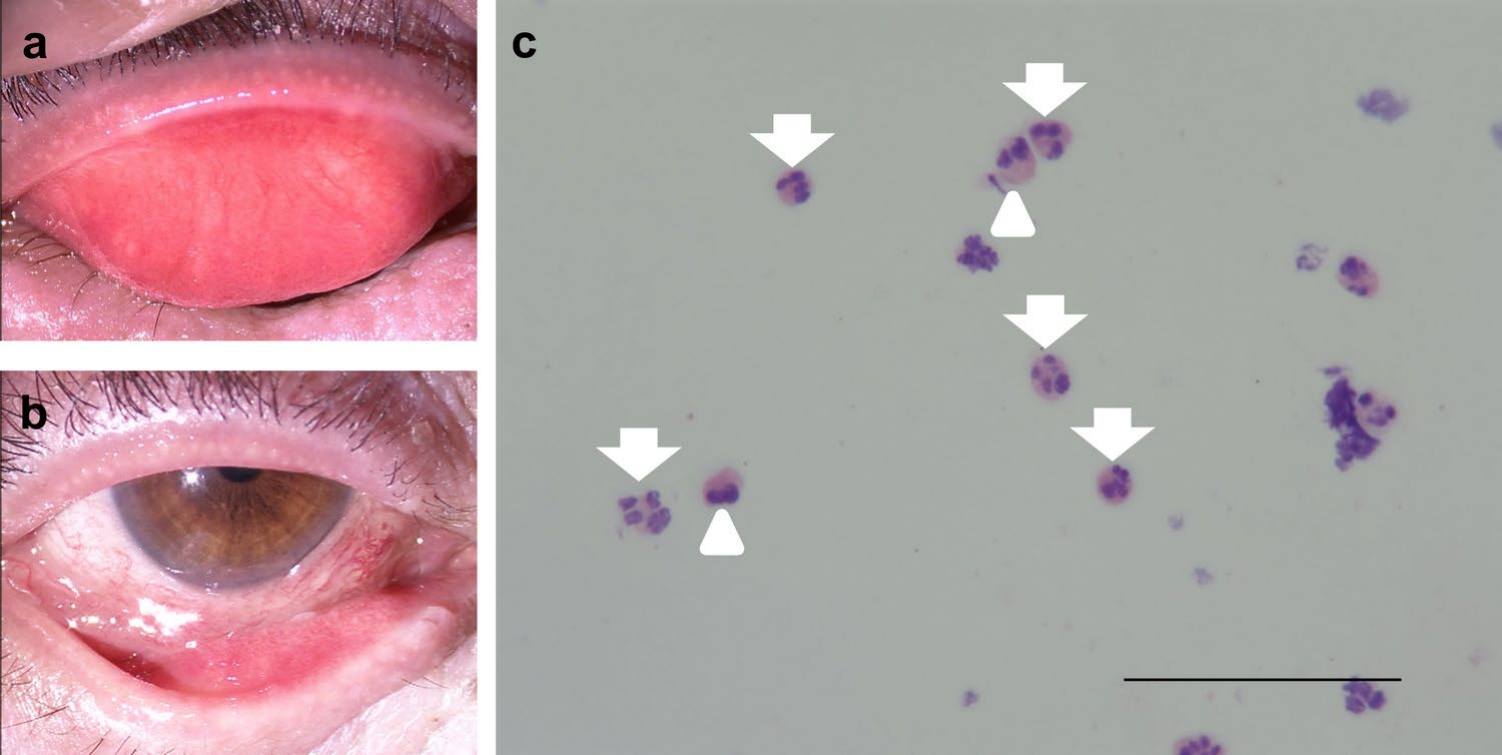
Conjunctival smear. Mixed cell infiltration of neutrophils and eosinophils observed in a conjunctival smear. **a, b** Photographs of slit-lamp examination in a patient with dupilumab-associated ocular surface disease (DAOSD). **c** Light-microscopic photograph of conjunctival smear in a patient with DAOSD. The smear results show a mixed inflammatory reaction caused by infiltration of both neutrophils (arrow) and eosinophils (arrowhead). Bar: 100 μm (×200)

Conjunctival smears are another important ophthalmologic examination for the diagnosis of DAOSD. They have shown neutrophil-dominant cell infiltration [87]. A significant conjunctival smear of neutrophils requires differential diagnosis with bacterial conjunctivitis. In patients with AD, however, eosinophils may be observed in the conjunctival smear, and a mixed inflammatory response of neutrophils and eosinophils may be observed (Fig. 7).

### Differential diagnosis

To diagnose DAOSD, it is necessary to differentiate it from acute exacerbations of AKC, dry eye associated with Sjögren syndrome, and psoriasis-associated conjunctivitis, which are Th2-, Th1-, and Th17-type conjunctivitis, respectively, as well as to differentiate it from infectious conjunctivitis. However, it is difficult to differentially diagnose these types of conjunctivitis on the basis of the clinical findings alone. Therefore, a proper diagnosis may require the assistance of ophthalmologic clinical examinations such as bacterial and viral cultures on the ocular surface, conjunctival smears, and biomarkers expressed in tears and on the ocular surface. Tear and ocular surface biomarker tests are expected to become important clinical tools in the future management of DAOSD.

## DAOSD management strategies

### General principles and treatment flow

The ophthalmologic treatment of DAOSD should be decided by taking the following into consideration: (1) DAOSD treatment is based on topical treatment of the eye using eye drops and ophthalmologic or dermatologic ointment applied to the eyelids. (2) The medications are selected according to the severity of DAOSD as determined by the clinical findings. (3) Unless the patient develops DAOSD that is too severe to treat, DAOSD treatment is administered while dupilumab treatment is continued. The incidence of DAOSD decreases to a low rate after 20 to 24 weeks of dupilumab administration [23], and some cases that require ophthalmologic treatment no longer require treatment after continuing dupilumab administration [3]. (4) Even in cases where treatment for DAOSD is successful, conjunctivitis can recur if treatment is discontinued after a short period of time.

### Treatment by severity

**Fig. 8:**
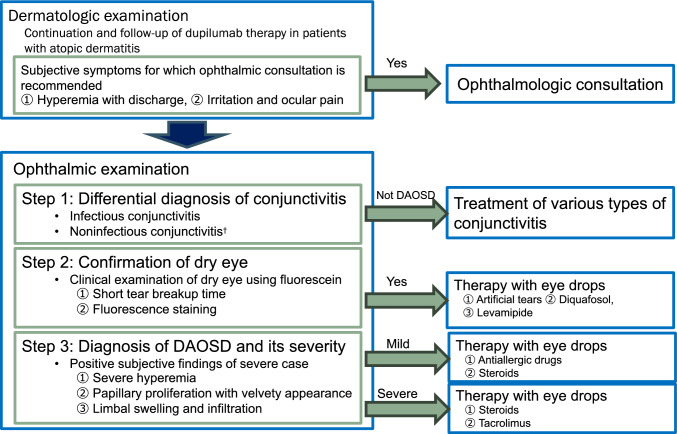
Japanese Society of Ocular Allergology management framework for dupilumab‐associated ocular surface disease (DAOSD). Dermatologists should consult an ophthalmologist if conjunctivitis symptoms, such as hyperemia and discharge, develop. An ophthalmologist should make a differential diagnosis of conjunctivitis, confirm the presence of dry eye, and determine the severity of DAOSD before selecting a therapeutic agent. ^†^Phlyctenular keratoconjunctivitis, Sjögren syndrome dry eye, ocular cicatricial pemphigoid, Stevens-Johnson syndrome, etc. *DAOSD* dupilumab-associated ocular surface disease

Treatment for mild DAOSD involves use of an ophthalmic solution of a histamine H_1_ receptor antagonist for symptoms of hyperemia and itching. In cases where dry eye is also present, artificial tears, rebamipide ophthalmic suspension, and diquafosol sodium ophthalmic solution may be used in combination (Fig. 8). Whilst artificial tears used before the onset of DAOSD are reported to have a preventive effect, treatments for dry eye, including artificial tear instillation, are not considered effective for severe cases of DAOSD [88, 89].

For severe DAOSD, topical steroids are used to treat severe conjunctivitis, blepharitis, and corneal infiltration (Fig. 8). The choice of steroid eye drops, such as fluorometholone, dexamethasone, and betamethasone, should depend on the severity of the DAOSD and the associated side effects. Recently, local administration of immunosuppressive drugs, including cyclosporine, tacrolimus, and pimecrolimus, has become an alternative DAOSD treatment to steroid eye drops [2, 5, 7, 88, 90, 91].

### Topical steroids and immunosuppressants

Ophthalmologic treatment for DAOSD is expected to occur over a long period of time, and because the long-term use of topical steroids increases the risk of complications such as steroid-associated glaucoma and cataract, these can only be considered for short term use, not for prolonged treatment [88]. As the effects of dupilumab become apparent, keratoconjunctivitis symptoms become less severe; therefore, the use of excessive steroid eye drops should be avoided [91]. Furthermore, the efficacy of topical steroids for the treatment of DAOSD is controversial. In the largest retrospective cohort study to date of patients with moderate-to-severe AD receiving dupilumab with long-term follow-up, steroid eye drops had a poor efficacy and may therefore not be optimal as a first-line treatment for DAOSD [3]. Additionally, other studies have recommended treatment of DAOSD with 0.1% fluorometholone ophthalmic solution, which has a low penetration rate into the eye and low risk of steroid side effects, or with 0.03% tacrolimus ophthalmic ointment [88]. Immunosuppressive eye drops are used alone or in combination with steroid eye drops. The effectiveness of immunosuppressive drug treatment has been confirmed in cases where cyclosporine eye drops were added to steroid eye drops for refractory cases of DAOSD and in cases treated with tacrolimus ointment eyelid application for severe blepharoconjunctivitis of DAOSD [2, 7, 91]. In Japan, tacrolimus ophthalmic suspension, which is indicated for VKC, may be an effective eye drop for treating DAOSD, although it remains as an off-label drug (Fig. 8) [86].

## International consensus and guidelines

### Red flag symptoms and referral criteria

Several institutions have published a consensus on the management of conjunctivitis that develops during dupilumab treatment in patients with AD. The International Eczema Council recommends consultation with an ophthalmologist at the onset of conjunctivitis and continued dupilumab treatment [92]. The International Eczema Council cited the fact that 68% of dermatologists strongly agree or agree with the statement “Patients with new onset conjunctivitis during dupilumab treatment should always be referred to an ophthalmologist” as a reason for recommending ophthalmologic intervention. The British Association of Dermatologists has published an expert consensus paper, which includes a review and recommendations aimed at supporting dermatologists and ophthalmologists in managing patients with DAOSD [5]. It states that since the major symptoms of DAOSD are conjunctivitis, dry eye, keratitis, and blepharitis, the diagnosis should be undertaken as per routine practice in ophthalmology. Ophthalmologic intervention is recommended when a patient treated with dupilumab presents with redness of the conjunctiva plus red-flag signs and symptoms, including worsening visual acuity (self-assessed), ocular pain, sensitivity to light, and visible damage to the cornea [5].

In addition, red-flag criteria have been established for indicating when dermatologists should request immediate ophthalmologic consultation. According to an Australian setting, the red flag indicating urgent ophthalmology referral is worsening or new onset of eye symptoms at any time during dupilumab therapy in patients with AD [93]. In Switzerland, the following 6 items have been established as red flags by consensus among specialists: vision loss, pain, purulent discharge, corneal involvement, conjunctival scarring, and contact lens wear [94].

### International and Japanese management frameworks

A management framework for the Australian setting recommended that the severity of subjective symptoms in patients with AD during dupilumab therapy should be evaluated with the DAOSD Activity Questionnaire (blurred vision, loss of vision, moderate-to-severe ocular redness, worsening/persistence of irritation, pain or light sensitivity, and severe mucopurulent discharge), and a sufficient severity requires review by an ophthalmologist to differentiate between DAOSD and other potential diagnoses [93]. In particular, concomitant keratoconjunctival diseases requiring urgent ophthalmologic consultation are bacterial keratitis, herpetic keratitis, and keratoconus.

Establishment of a common understanding of ophthalmic daily practice for DAOSD is essential to continue dupilumab therapy with ophthalmologic intervention. Furthermore, because DAOSD is a highly variable disease, a management framework is important for creating a treatment plan based on the ophthalmologic findings. A management framework in Japan was proposed by the Case Study Committee on IL-4- and IL-13-associated monoclonal antibody therapy (CSC4.13) of the Japanese Society of Ocular Allergology, on the basis of a questionnaire survey among ophthalmologic and allergologic specialists (Fig. 8) [95]. The first key point of this management framework was clinical collaboration between ophthalmologists and dermatologists, who should recommend ophthalmologic consultation when conjunctivitis symptoms appear. The second key point was that after evaluating the subjective and objective symptoms of patients undergoing dupilumab therapy who visit an ophthalmologist, the ophthalmologist should make a diagnosis and decide on a treatment plan on the basis of the following: (1) A differential diagnosis of conjunctivitis other than DAOSD. (2) Confirmation of the presence of absence of dry eye. (3) Determination of DAOSD severity based on the ophthalmologic findings including conjunctival hyperemia, papillary proliferation in the palpebral conjunctiva, limbal swelling, and cellular infiltration or white dots in the peripheral cornea.

In the future, ophthalmologists will be expected to manage DAOSD appropriately through collaboration between ophthalmology and dermatology departments and with ophthalmologic intervention. The development of clinical tests for ophthalmology using biomarkers may also become an indispensable tool for improving DAOSD ophthalmic practice.

## Conclusion

In conclusion, severe cases of DAOSD require ophthalmologic intervention, and clinical collaboration between ophthalmologists and dermatologists is crucial for patients with AD during dupilumab treatment. By presenting an overview of the knowledge on the clinical characteristics and pathogenesis of DAOSD and by highlighting recommendations for its management and treatment, this review can assist the daily practice of ophthalmologists in Japan.
